# Upregulation of HCN2 in ventral tegmental area is involved in morphine‐induced conditioned place preference in rats

**DOI:** 10.1002/2211-5463.13888

**Published:** 2024-09-12

**Authors:** Jie Yin, Yang Li, Dan Li, Chenxu Chang, Xiechuan Weng

**Affiliations:** ^1^ Department of Neuroscience Beijing Institute of Basic Medical Sciences China; ^2^ Department of Experimental Pathology Beijing Institute of Radiation Medicine China; ^3^ Jingnan Medical Area of the General Hospital of the People's Liberation Army Beijing China

**Keywords:** addiction, conditioned place preference, current, HCN channel, morphine, mRNA

## Abstract

Morphine is an opioid commonly used to treat pain in clinic, but it also has the potential to be highly addictive, which can lead to abuse. Despite these known risks, the cellular and molecular mechanism of morphine conditioned place preference (CPP) is still unclear. In this study, using a rat model of chronic morphine administration, we found that compared with the control group, the mRNA and protein expression of HCN2 channel in the ventral tegmental area (VTA) were upregulated. Further immunofluorescence analysis showed that the fluorescence intensity of HCN2 channel of VTA dopaminergic neurons in morphine group was significantly enhanced, while the patch clamp recording of brain slices showed that both the magnitude and the density of *I*_h_ (HCN channel current) of VTA neurons were significantly increased. Moreover, intra‐VTA infusion of ZD7288, a selective inhibitor of HCN channel, into rats of the morphine group decreased morphine CPP. Taken together, our results show that chronic morphine administration induces an upregulation of HCN2 in VTA dopamine neurons, while HCN inhibition reduces morphine CPP, suggesting that HCN channel may be a potential target for the treatment of morphine addiction.

## Graphical abstract

The cellular and molecular mechanism of morphine conditioned place preference (CPP) is still unclear. Our findings suggest that the upregulation of HCN2 in VTA induced by chronic morphine administration contributes to morphine CPP. Interestingly, this study may provide new mechanisms and intervention clues for the treatment and intervention of morphine addiction.

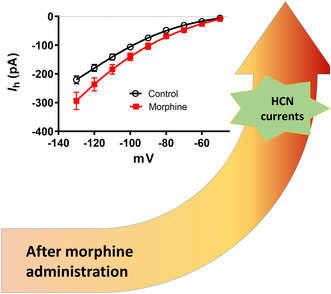

**Abbreviations**
4‐AP: 4‐aminopyridine
ACSF: artificial cerebrospinal fluid
CPP: conditioned place preference
HCN channel: hyperpolarization‐activated cyclic nucleotide‐gated channel
*I*_h_
: HCN currents
PVDF: polyvinylidene difluoride
RIPA: radioimmunoprecipitation assay
TEA: tetraethylammonium
TTX: tetrodotoxin
VTA: ventral tegmental area

Morphine is a powerful analgesic and widely used in clinical treatment. It mainly produces analgesic effect by activating μ‐opioid receptors in the central nervous system [1, 2, 3, 4]. As an analgesic drug, morphine has potential risk of abuse and addiction [5]. Studies have found that dopamine reward neural pathway and dopaminergic neurons in ventral tegmental area (VTA) play key role in the process of addiction, and conditioned place preference (CPP) induced by methamphetamine and cocaine [6, 7, 8, 9]. However, the role and molecular mechanism of dopaminergic neurons in VTA in morphine‐induced addiction and CPP formation are still unclear.

Hyperpolarization‐activated cyclic nucleotide‐gated channels (HCN channels) exist in tissues and cells such as central and peripheral nerves, cardiac rhythm cells, and retinal photoreceptors. HCN channel is a tetramer composed of four subtypes HCN1, HCN2, HCN3, and HCN4 in different combinations. The subunits of HCN channel are different in electrophysiological dynamics, sensitivity to cAMP, and distribution in different types of neurons. HCN channel is activated during hyperpolarization, and its equilibrium potential is slightly higher than the resting membrane potential of cells. Its special electrophysiological characteristics make it play an important role in maintaining the stability of neuronal membrane potential, which is closely related to the excitability of cells and plays an important role in pain, addiction, and epilepsy [10, 11].

HCN channel is closely related to addiction. Recent studies have found that HCN channels in VTA are involved in the formation of cocaine addiction [12, 13, 14]. Cocaine addiction can increase the expression of HCN channels in prefrontal cortex, VTA, and other brain regions, while another study pointed out that the expression of HCN2 and HCN4 channels in VTA were downregulated after cocaine addiction. Although the observed results on the expression of HCN channel subtypes were different in addiction and the involved mechanism remained unclear, these studies suggested that HCN channels were related to cocaine addiction [13, 14]. In addition, HCN channels in nucleus accumbens were found to participate in the positive reinforcement of methamphetamine and the motivation of drug use. ZD7288, a selective blocker of HCN channel, could significantly reduce the excitability and positive reinforcement, as well as the contextual learning caused by methamphetamine, and regulate the self‐administration behavior of rats [9].

Previous studies showed that chronic morphine exposure (subcutaneous administration for 7 days) significantly inhibited the long‐term potentiation of hippocampal CA1 area, significantly decreased the expression of HCN1 and increased the expression of HCN2, and impaired learning and memory [15]. Through gene chip screening, we found that the expression of HCN2 channel in four subtypes of HCN channels in VTA of rat changed after chronic morphine administration. However, the function and role of HCN channels as well as their subtypes in VTA in morphine addiction and dependence are still unknown.

Here, we established a morphine addiction model in rats by continuous intraperitoneal injection of morphine for 14 days. RT‐PCR and western blot were used to analyze the changes in HCN channel expression in VTA neurons of morphine‐addicted rats, and the changes in HCN2 expression in VTA dopaminergic neurons were observed by immunofluorescence. The changes in HCN channel current in VTA neurons after morphine administration were further determined by electrophysiological experiments. Finally, we observed the effects of HCN channel blocker on morphine‐induced CPP. Therefore, the purpose of this study was to explore the expression change and function of HCN channel in chronic morphine administration and its role in the formation of morphine CPP.

## Materials and methods

### Animals

**Fig. 1:**
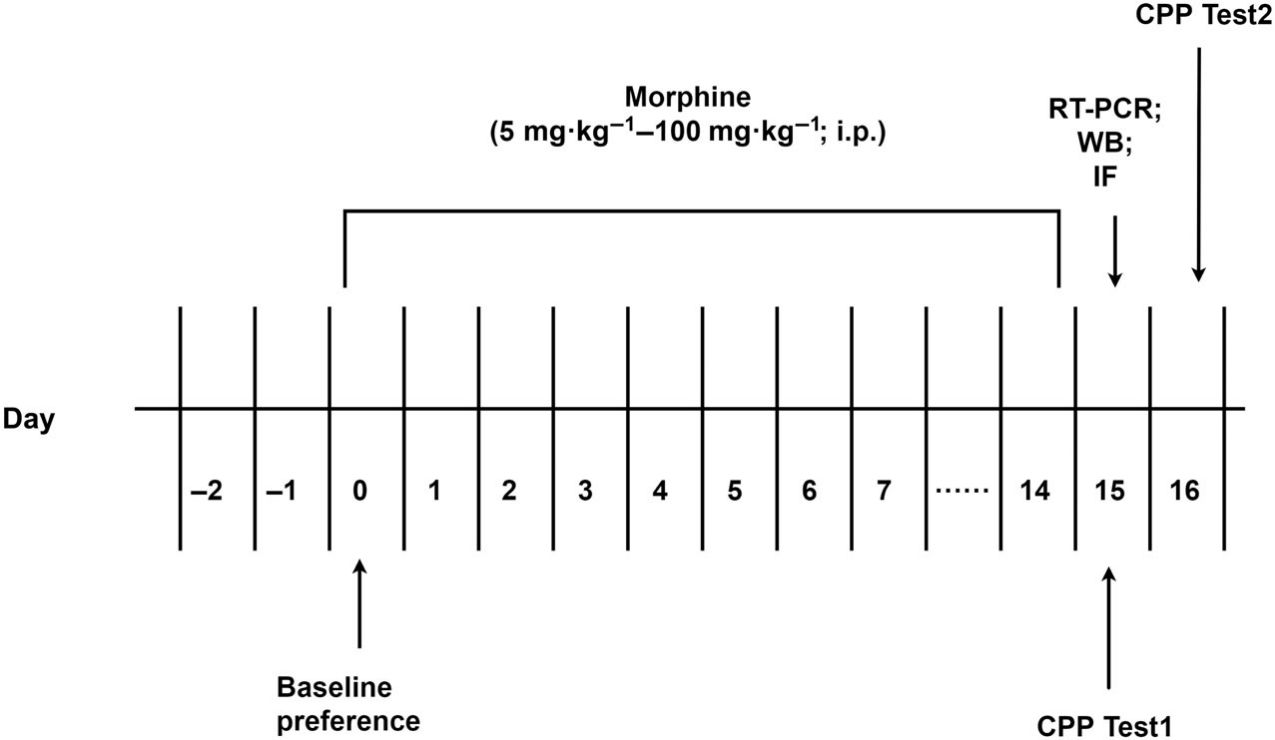
Time line of the experiments.

Sprague–Dawley rats (5 weeks, 180–200 g) were procured from SPF Biotechnology Co., Ltd. (Beijing, China), which were randomly divided into morphine group and control group (13 females and 13 males in each group) in a temperature‐controlled environment (21–22 °C), within an animal holding room, following a 12‐h light/12‐h dark cycle (lights on at 7:00 am). For each group of 26 rats, six rats were used for RT‐PCR and WB experiments, five rats were used for immunofluorescence experiments, five rats were used for electrophysiological recording, and the remaining 10 rats were used for the second CPP experiment (CPP Test 2) (Fig. 1). Food and tap water were available *ad libitum*. Experiments were performed in compliance with the National Institutes of Health Guide for the Care and Use of Laboratory Animals. After experiments, the remaining rats were euthanized in a CO_2_‐filled chamber. The Institutional Animal Care and Use Committee at the Animal Center (Beijing, China) sanctioned the experimental protocol (Approval No. IACUC‐DWZX‐2022‐715).

### Drug administration

For the rat model of chronic morphine administration, morphine hydrochloride (Shenyang First Pharmaceutical Factory, Shenyang, Liaoning, China) was dissolved in saline and administered intraperitoneally (i.p.) by increasing the dose of morphine for 14 consecutive days [10, 16, 17, 18, 19, 20]. The initial dose of morphine group was 5 mg·kg^−1^ on the first day, and the dose gradient was 5 mg·kg^−1^·day^−1^ from Day 2 to Day 7, 10 mg·kg^−1^·day^−1^ from Day 8 to Day 14, and reached 100 mg·kg^−1^ on Day 14. Morphine is administered twice a day at 8:00 am and 8:00 pm with an interval of about 12 h [14]. The control group was injected with the same volume of saline according to body weight.

### HCN channel blocker ZD7288 was infused into the VTA of rats

Rats in morphine group were anesthetized by intraperitoneal injection of 2% sodium pentobarbital (0.1 g·kg^−1^). After anesthesia, the rats were fixed to the brain stereotaxic apparatus and the anterior fontanelle was exposed. The anterior fontanel point was used as the reference point of the three‐dimensional coordinate system, and the VTA position was determined according to the stereotaxic map of the brain (AP: −5.2 mm, ML: ±1.8 mm, DV: 8.0 mm), the injection catheter was placed and secured with denture cement. The infused VTA was located according to the coordinates [21]. ZD7288 (TargetMol Chemicals Inc., Boston, MA, USA) was infused slowly (5 μg·kg^−1^, 0.05 μL·min^−1^) into the VTA, and the injection catheter was allowed to remain in the VTA for 3 min.

### The CPP experiment

A conditional location preference apparatus was utilized, comprising two compartments (30 × 60 × 30 cubic centimeters; Beijing Zhong Shi Di Chuang Science and Technology Development Co., Ltd, Beijing, China). The compartments had distinct floors and walls, separated by a removable plastic board. The walls and floor of one compartment were black, whereas those of the other compartment were white. A video camera mounted above the device allowed observation of each rat's behavior on a video monitor [22, 23]. Rats were allowed to freely explore both sides of the CPP apparatus for 15 min to assess their baseline place preference (Baseline preference) on Day 0. The average time spent in each compartment was recorded for both groups of rats. The white compartment was designed as the morphine administration compartment. By calculating the time difference between the two sides of the rats, the rats with < 200 s entered the next stage.

From Day 1 to Day 14, the rats in the morphine group were injected with morphine and confined to white chamber for 45 min, and then injected saline and confined to black chamber for 45 min. Rats in the control group received saline injection in both chambers. On Day 15, 40 rats were randomly selected for CPP Test 1, with 20 rats in each group, including 10 females and 10 males. The rats were placed in the middle of the CPP apparatus and allowed to explore the two chambers freely for 15 min. The time spent in the white chamber was recorded (CPP Test 1). On Day 16, the remaining 20 rats (10 rats in each group, 5 males and 5 females) were conducted the CPP Test 2 after ZD7288 was infused into the VTA (Fig. 1).

### Extraction and quantification of mRNA from VTA for PCR assays

A total of 12 rats were used in RT‐PCR and western blot experiments (six rats in each group, three females and three males). After the rats were sacrificed under anesthesia, the brain tissue of the rats was removed and stored in a −20 °C refrigerator on ice for subsequent analysis. Total RNA was extracted from frozen midbrain tissue by RNAiso reagent (Servicebio, Wuhan, China) and reverse transcribed using SweScript All‐in‐One SuperMix for qPCR (Servicebio). Next, engineered gene expression was quantified using Universal Blue SYBR Green qPCR Master Mix (Servicebio) on a PCR machine (Thermo Fisher Scientific, Shanghai, China). One is gene expression from GAPDH genetic engineering. Engineered primers and GAPDH‐specific primers were obtained from Servicebio and described as follows: HCN1 primers: 5′‐CATACTGTCGCCTTTACTCCCTT‐3′ (forward), 5′‐TAGAGTTTTTCTTGCCTATCCGATC‐3′ (reverse); HCN2 primers: 5′‐CGCCACCTGCTACGCTATGTT‐3′ (forward), 5′‐TCCACTTGCTTGTACTTCTCCTG‐3′ (reverse); HCN3 primers: 5′‐GTTGGAGAGGCCCAACGAGT‐3′ (forward), 5′‐CGATTTCCACCGCTTTGTGG‐3′ (reverse); HCN4 primers: 5′‐TCCTATTTTGGAGAGATCTGCTTG‐3′ (forward), 5′‐AGCGCAACCGTCTCGAAAG‐3′ (reverse); GAPDH: 5′‐CTGGAGAAACCTGCCAAGTATG‐3′ (forward), 5′‐GGTGGAAGAATGGGAGTTGCT‐3′(reverse). Gene expression was analyzed using the standard ΔΔ*C*_q_ method.

### Protein extraction and western blot

After the rats (six rats in each group, three males, and three females) were sacrificed under anesthesia, the middle brain tissue was removed and homogenized in radioimmunoprecipitation assay (RIPA) lysis buffer with protease inhibitor PMSF for 1 h at 4 °C. Protein concentrations were analyzed using the BCA protein assay kit (Servicebio). Protein samples were electrophoresed on SDS/PAGE gels and transferred to polyvinylidene difluoride (PVDF) membranes. Then, the membranes were blocked with 5% skim milk for 2 h at room temperature and sequentially incubated overnight at 4 °C with primary antibodies (HCN2, dilution ratio 1 : 1000, Cat No. APC‐030, Alomone Lab, Jerusalem, Israel; GAPDH, dilution ratio 1 : 3000, Cat No. GB15004, Servicebio) diluted in TBST. The next day, the secondary antibody (HRP Goat Anti‐ Mouse IgG, dilution ratio 1 : 3000, Cat No. GB23301, Servicebio; HRP Goat Anti‐Rabbit IgG, dilution ratio 1 : 3000, Cat No. GB23303, Servicebio) was used for 1 h at 37 °C. Then, the membrane was visualized in the imaging system using the ECL kit (Servicebio). GAPDH was used as a control to normalize protein expression.

### Immunofluorescence

After anesthesia, five rats in the morphine and control groups (three females and two males in each group) were fixed by cardiac perfusion with PBS and 4% paraformaldehyde. Brain tissues were then removed and fixed in 4% paraformaldehyde. VTA brain samples (10 μm) were extracted from Thermo Fisher Scientific, CRYOSTAR NX50, USA by stereotaxic of rat brain. Frozen sections were stored in phosphate buffer solution (PBS; pH 7.4) after washing, the cells were preincubated with PBS blocking solution containing 1% bovine serum albumin and 4% normal goat serum for 1 h at room temperature. The sections were then treated with tyrosine hydroxylase (TH) monoclonal antibody (dilution ratio 1 : 400; Servicebio) and HCN2 channel monoclonal antibody (dilution ratio 1 : 200; Alomone Lab). The cells were incubated for 24 h in PBS containing 2% normal goat serum. After washing, sections were incubated at room temperature in the dark with Alexa 488‐conjugated secondary antibodies (goat anti‐rabbit antibody; IgG; Servicebio) 1 : 400 diluted and Alexa 594‐conjugated secondary antibody (goat anti‐mouse antibody; IgG; Jackson, West Grove, PA, USA) in 1 : 400 diluted PBS for 2 h. The sections were then washed three times for sealing. Sections were observed under a fluorescence microscope (Nishi‐Ōi, Shinagawa, Tokyo, Japan).

The fluorescence intensity of DAPI and HCN2 channel in the visual field with TH staining positive in VTA was analyzed by the halo image analysis software (Indica labs, Albuquerque, NM, USA). Sections with no statistically significant difference in DAPI fluorescence intensity were selected, and the expression level of HCN2 channel was evaluated by fluorescence intensity.

### Slice preparation and electrophysiology

Five rats in the morphine and control groups (two females and three males in each group) were anesthetized by inhalation of isoflurane and decapitated to obtain brain tissue. Sections (mm) were prepared in frozen section solution containing: 5 KCl, 1.25 NaH_2_PO_4_, 26 NaHCO_3_, 212.7 sucrose, 180.2 glucose, 1 CaCl_2_, and 1 MgCl_2_. The sections of the VTA were cut according to the stereotaxic map of the rat brain using a vibration microtome (VT1200; Leica, Germany). ACSF was prefilled in an incubator and placed in a thermostable water bath at 32 °C for half an hour. Slices were recovered in ACSF for 30 min prior to patch‐clamp electrophysiological recording of brain slices. The above procedures were performed in continuously saturated 95% O_2_ and 5% CO_2_.

Data were acquired using an Axoclamp patch clamp amplifier (700B; Axon, San Jose, CA, USA) and a DigiData digitizer (1550B; Axon) and analyzed using Clampfit (Axon). Data were filtered at 2 kHz and collected at a 10 kHz sampling rate. Action potential (AP) firing was recorded in whole‐cell mode, and fast and slow capacitance compensation was performed after tight seals (> 1 GΩ) were formed. The NaHCO_3_‐buffered saline of *I*_h_ was recorded as (mm): 115 NaCl, 5 KCl, 1.25 NaH_2_PO_4_, 25 NaHCO_3_, 10 sucrose, 2 sodium pyrurate, 2 CaCl_2_, 2 MgCl_2_, pH 7.4. BaCl_2_ (1 mm) and TTX (0.5 μm) were added to saline to block inward rectified K^+^ and Na^+^ channels, respectively. CdCl_2_ (0.1 mm), 4‐AP (2 mm), and TEA (5 mm) were also added to saline to block voltage‐dependent Ca^2+^ and K^+^ channels. In the voltage‐clamp model, *I*_h_ was activated by a hyperpolarizing voltage from −50 to −130 mV. To ensure the stability of whole‐cell recordings, the time interval from scan start to start was 5 s. Series resistance was monitored during all recordings, and data were discarded if resistance changed by more than 25% or > 50 MΩ [12, 24, 25, 26, 27].

### Statistical analysis

Statistics are expressed as mean ± SEM. Data were analyzed using prism graphpad (GraphPad Software, Boston, MA, USA) using two‐way ANOVA with Bonferroni correction and Wilcoxon rank sum test. In data processing, *P* < 0.05 was considered statistically significant.

## Results

### Upregulation of HCN2 expression in VTA induced by chronic morphine administration

**Fig. 2:**
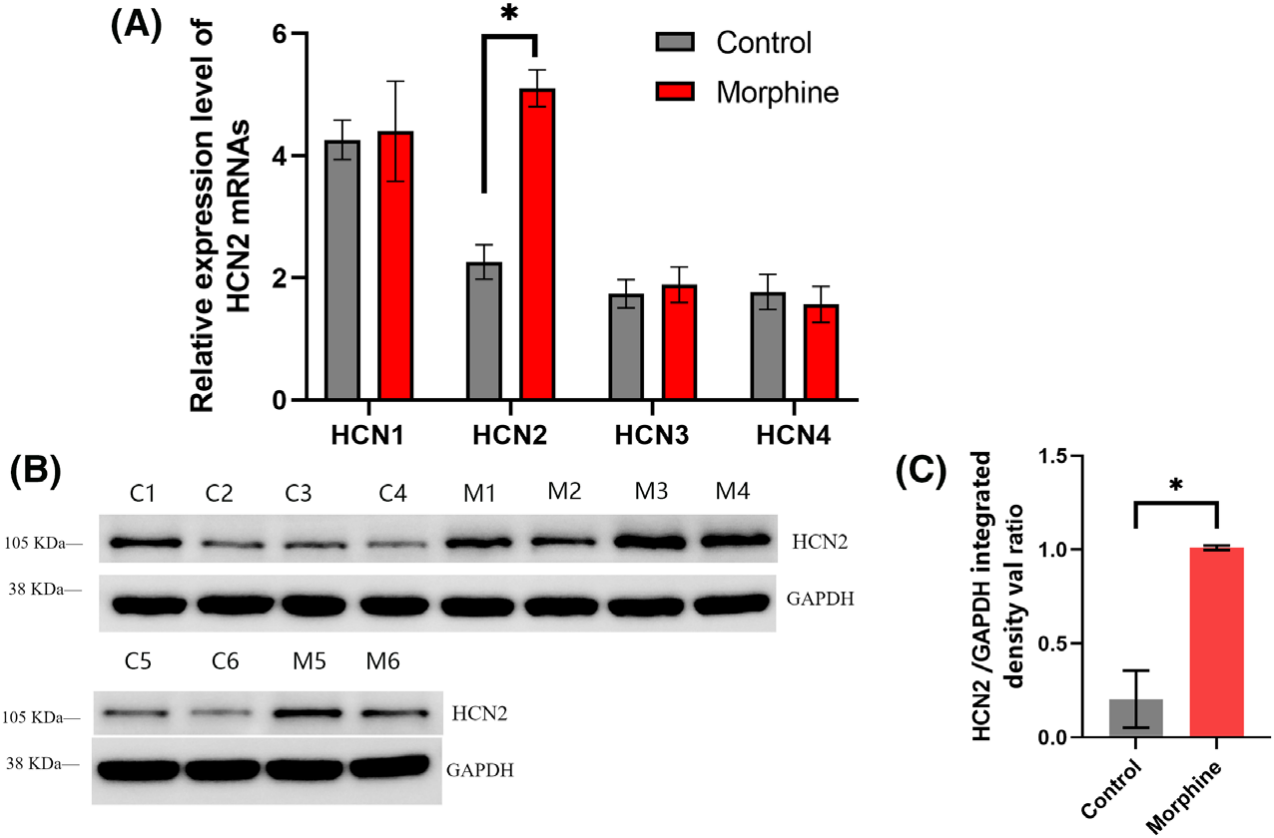
Upregulation of HCN2 expression in VTA induced by chronic morphine administration. (A) The mRNA expression levels of HCN1, HCN3 and HCN4 in morphine group (*n* = 6) were not significantly different from that in control group (*P* = 0.38 > 0.05), while HCN2 was significantly higher than that in control group (*n* = 6, *P* = 0.028 < 0.05). (B) Morphine group (M1–6) showed significantly stronger blots than control group (C1–6). (C) The expression level of HCN2 channel protein (relative gray value). By quantitative analysis, the expression level of HCN2 channel protein of morphine group (*n* = 6) was significantly higher than that of control group (*n* = 6, *P* = 0.0012 < 0.05). The results were analyzed using Wilcoxon rank sum test. The error bars indicate SEM. *P < 0.05.

Through quantitative analysis of RT‐PCR *C*_t_ value and conversion of amplification fold by ΔΔ*C*_t_ method, statistical analysis showed that there was no significant difference in the mRNA expression level of HCN1, HCN3, and HCN4 gene between morphine group and control group (*n* = 6, *P* = 0.38 > 0.05, Fig. 2A) and the mRNA level of HCN2 gene expression in morphine group was significantly higher than that in control group (*n* = 6, *P* = 0.028 < 0.05, Fig. 2A). And we did not find obvious difference in HCN2 gene expression between rats of different gender in the morphine group (*n*_F_ = 3, *n*_M_ = 3, *P* = 0.67 > 0.05). In addition, the expression of HCN2 channel protein in morphine group (M1–6) and control group (C1–6) was studied by western blot (Fig. 2B). Through further quantitative analysis, it showed that the gray value of the western blot band of HCN2 protein in the morphine group was significantly upregulated from that in the control group (*n* = 6, *P* = 0.0012 < 0.05, Fig. 2C). And there was no significant difference in the gray value of the western blot band of HCN2 protein between male and female in morphine group (*n*_F_ = 3, *n*_M_ = 3, *P* = 0.41 > 0.05).

With RT‐PCR and western blot experiments, it was found that both the expression levels of HCN2 mRNA and protein in the VTA of morphine group were significantly increased compared with the control group.

### Increase of HCN2 immunofluorescence intensity of dopaminergic neurons in VTA

**Fig. 3:**
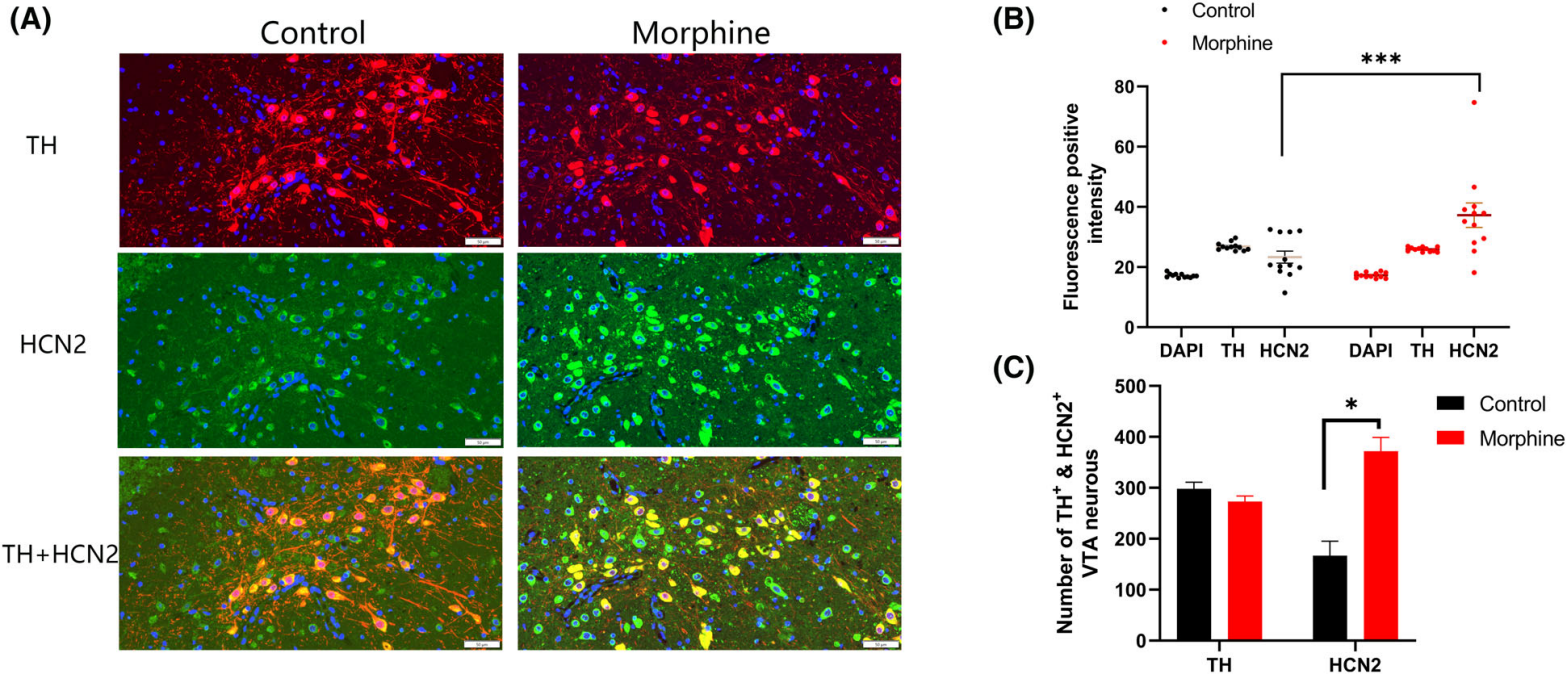
Immunofluorescence intensity of HCN2 and TH in VTA. (A) The TH, HCN2, and TH & HCN2 IF staining in the VTA of the control group and the morphine group (*n* = 5 rats per group). The fluorescence intensity of HCN2 channel of dopaminergic neurons in the morphine group was significantly stronger than that in the control group, scale bar = 50 μm. (B) The IF of DAPI, TH and HCN2 analysis between the control group and the Morphine group. For the TH^+^ VTA neurons, the fluorescence intensity of HCN2 in the morphine group was significantly higher than that in the control group (*n* = 5 rats per group, *P* = 0.0003 < 0.001). (C) The number of VTA TH^+^ and HCN2^+^ neurons. Comparing the green fluorescence intensity and the number of HCN2 of the dopaminergic neurons with red staining of TH, the morphine group was significantly higher than that of the control group (*n* = 12 imaged sections from 5 rats per group, *P* = 0.017 < 0.05). Besides, the number and fluorescence intensity of TH‐labeled dopaminergic neurons in the VTA were not significantly different from those in the control group (*n* = 12 imaged sections from 5 rats per group, *P* = 0.45 > 0.05). The results were analyzed using Wilcoxon rank sum test. The error bars indicate SEM. *P < 0.05, ***P < 0.001.

Tyrosine hydroxylase (TH) red fluorescence staining was used to assist the localization of dopaminergic neurons in the VTA, and HCN2 green fluorescence staining indicated the HCN2 channel in the VTA. For the dopaminergic neurons with red staining of TH (Fig. 3A), the IF intensity of HCN2 in the morphine group (*n* = 5) was significantly higher than that in the control group (*n* = 5). And we did not find obvious gender differences between male and female rats in each group (two females and one male rats for each group).

After further quantitative measurement of the green fluorescence staining intensity of HCN2 in the figure, under the condition that there was no difference in the intensity of blue DAPI staining in the nucleus, it could be considered that the fluorescence intensity of different IF sections was comparable, and the experimental data were statistically analyzed. Through the analysis of the IF intensity of the two groups in Fig. 3B, there is no statistically significant difference in the IF intensity of DAPI between the two groups. And for the dopaminergic neurons with red staining of TH, the fluorescence intensity of HCN2 in the morphine group was significantly higher than that in the control group (*n* = 5 rats per group, *P* = 0.0003 < 0.001), and the number of HCN2‐expressed (HCN2^+^) neurons in the VTA in the morphine group was significantly increased compared with the control group (*n* = 5 rats per group, *P* = 0.017 < 0.05, Fig. 3C). The percentage of VTA neurons expressing HCN2 and TH in the control group were 27.41% and 47.46%, respectively (HCN2: 2010 of 7332 neurons, 27.41%; TH: 3480 of 7332 neurons, 47.46%), while they were 64.12% and 46.94%, respectively, in morphine group (HCN2: 4463 of 6960 neurons, 64.12%; TH: 3267 of 6960 neurons, 46.94%) (*n* = 12 imaged sections from five rats per group). In addition, there was no significant difference for the TH fluorescence intensity in the VTA neurons between morphine group and control group (*n* = 5 rats per group, *P* = 0.45 > 0.05).

### *I*_h_ in VTA neurons were altered by chronic morphine administration

**Fig. 4:**
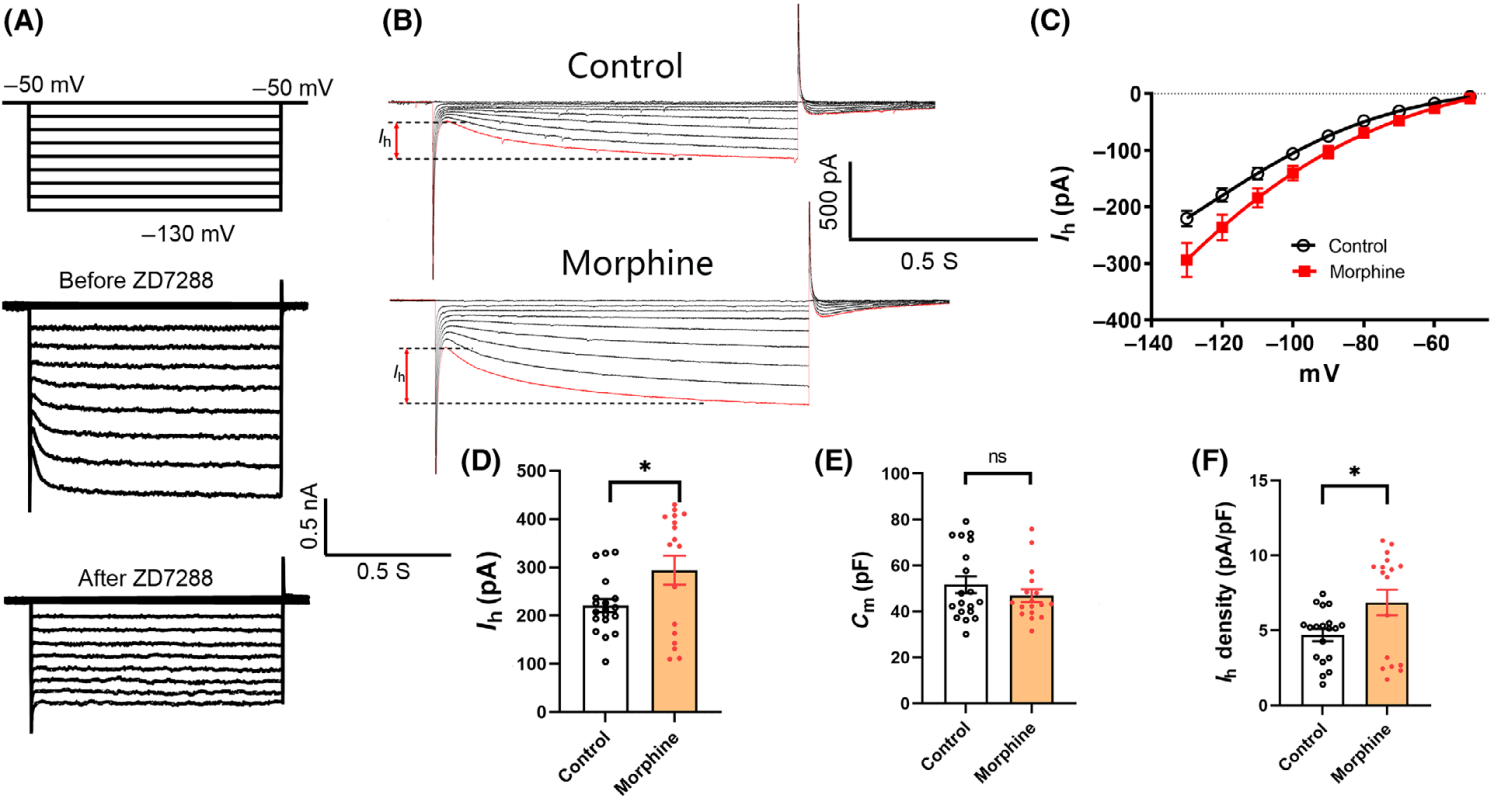
*I*_h_ were increased after chronic morphine administration. (A) Voltage protocol from −50 to −130 mV for recording *I*_h_ current. *I*_h_ were blocked by ZD7288 (50 μm). (B) HCN channel current *I*_h_ in morphine group and in control group. (C) The *I*_h_ in both the control group (*n*_c_ = 19) and the morphine group (*n*_m_ = 17) showed current–voltage dependence. (D) The average *I*_h_ in morphine group at −130 mV was significantly larger than that of the control group (*n*_c_ = 19, *n*_m_ = 17, *P* = 0.028 < 0.05). (E) The membrane capacitance (*C*_m_) was no significantly difference between control group and morphine group (*n*_c_ = 19, *n*_m_ = 17, *P* = 0.31 > 0.05). (F) The average *I*_h_ density in morphine group at −130 mV was significantly larger than that of the control group (*n*_c_ = 19, *n*_m_ = 17, *P* = 0.022 < 0.05). The results were analyzed using Wilcoxon rank sum test. The error bars indicate SEM. *P < 0.05.

HCN currents (*I*_h_) were recorded by patch‐clamp technique in rat brain slices. In the experiment, voltage stimulation from −50 to −130 mV was used, and the recorded current could be inhibited by HCN channel specific blocker ZD7288 (50 μm) (Fig. 4A). HCN channel currents were successfully recorded in 19 neurons of the control group and 17 neurons of the morphine group. Compare with the control group (*n* = 19), the amplitude of HCN channel current (*I*_h_) increased significantly in morphine group (*n* = 17) (Fig. 4B). The *I*_h_ in both the control group and the morphine group showed current–voltage dependence (Fig. 4C). The average current amplitude of *I*_h_ in morphine group (293.93 ± 30.17 pA, *n*_m_ = 17) was 33.27% higher than that in control group (220.56 ± 13.77 pA, *n*_c_ = 19) (*P* = 0.028 < 0.05, Fig. 4D). There was no significant difference in membrane capacitance (*C*_m_) between control group (51.67 ± 3.61 pF, *n*_c_ = 19) and morphine group (46.88 ± 2.81 pF, *n*_m_ = 17) (*P* = 0.31 > 0.05, Fig. 4E). The average current density of *I*_h_ in morphine group (4.69 ± 0.40 pA·pF^−1^, *n*_m_ = 17) was 46.48% higher than that in control group (6.87 ± 0.85 pA·pF^−1^, *n*_c_ = 19) (*P* = 0.022 < 0.05, Fig. 4F).

### *I*_h_ blocker ZD7288 inhibited morphine CPP

**Fig. 5:**
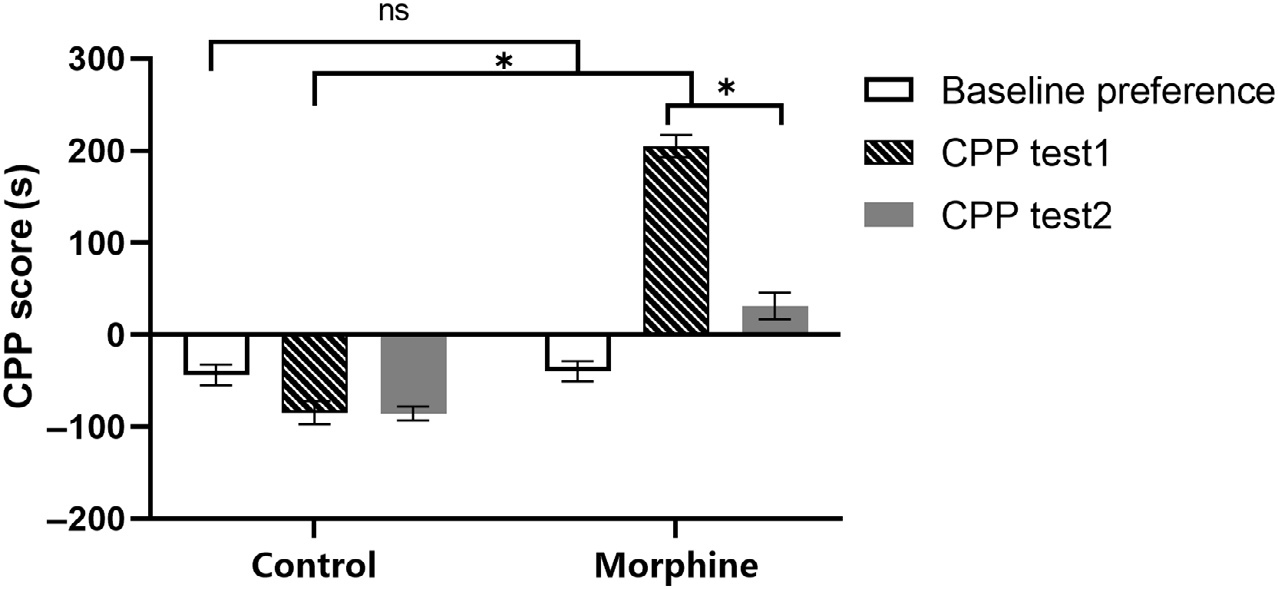
Conditioned place preference (CPP) before and after chronic morphine administration. Before morphine administration (on Day 0), there was no significant difference in the CPP Score of baseline preference between morphine group and control group (*n* = 10, *P* = 0.64 > 0.05). After chronic morphine administration (on Day 15), the CPP Score increased significantly in morphine group compare with the control group (CPP Test 1) (*n* = 10, *P* = 0.03 < 0.05). After ZD7288 infusion in VTA (on Day 16), the morphine CPP in morphine group was inhibited compare with that of Day 15 (CPP Test 2) (*n* = 10, *P* = 0.01 < 0.05). The results were analyzed using two‐way ANOVA with Bonferroni correction. The error bars indicate SEM. *P < 0.05.

Conditioned place preference were conducted for morphine group and the control group on Day 0 (baseline preference), Day 15 (CPP Test 1), and Day 16 (CPP Test 2). For the CPP analysis, a two‐way ANOVA followed by Bonferroni correction was performed. Figure 5 shows the results before chronic morphine administration (baseline preference), showing no statistically significant difference between the morphine (*n* = 20) and control groups (*n* = 20) (*P* = 0.64 > 0.05). For the CPP Test 1 after chronic morphine administration (Day 15), there was a significant difference in the CPP Score between the morphine group and the control group (*n* = 20, *P* = 0.03 < 0.05), suggesting that morphine increased the CPP Score compared with saline.

For the morphine group, the CPP Score on Day 15 (Before ZD7288) was significantly longer than that of Day 16 (After ZD7288) (*n* = 10, *P* = 0.01 < 0.05), which suggested that HCN channels blocker ZD7288 inhibited morphine‐induced CPP.

## Discussion

As a commonly used analgesic in clinic, it is of great significance to clarify the cellular and molecular mechanism of morphine addiction [28]. CPP is closely related to drug addiction, and it is also one of the important indicators to evaluate addiction. CPP experiment can be used to evaluate drug reward effect and animal‐related learning and memory, and it is a classic model to study drug addiction. In this study, we systematically studied whether CPP induced by chronic morphine administration is related to HCN channels of VTA neurons. Through gene chip screening, we found that the expression of HCN2 channel in four subtypes of HCN channel in ventral tegmental area of midbrain in rats with chronic morphine administration was upregulated. Thus, we used RT‐PCR and western blot to verify that the mRNA expression of HCN2 channel in VTA of rats with chronic morphine administration was upregulated and the expression of HCN2 protein was increased. Immunofluorescence study showed that the fluorescence intensity of HCN2 channel in dopaminergic neurons in VTA was significantly enhanced after morphine administration. These results showed that chronic morphine administration induced the increase of HCN2 expression in dopaminergic neurons of VTA in rats, and there were no obvious gender differences between male and female rats in control group and morphine group. In the following study, we found that the *I*_h_ altered significantly after morphine was given chronically, and blocking the HCN current could reduce morphine CPP. These results indicate that HCN2 of VTA neurons is involved in morphine addiction, and HCN2 may be a potential target for the treatment of morphine addiction.

Drug addiction involves chronic encephalopathy with pathological changes in neural circuits, and the reward pathway formed by VTA projection to dorsal striatum is the crucial neurophysiological basis of drug addiction. HCN channel of VTA dopaminergic neurons is involved in regulating neuronal discharge activity and neuronal excitability and plays an important role in the occurrence of drug addiction [29, 30, 31, 32, 33]. Recent studies have found that the HCN channel of reward pathway plays a key role in the positive reinforcement effect and motivation of methamphetamine. The HCN channel blocker ZD7288 can obviously reduce the excitability and positive reinforcement effect of methamphetamine and regulate the self‐administration behavior of rats [9]. In addition, cocaine could induce upregulation of HCN channel in VTA dopamine neurons, while inhibition of HCN can reduce the motivation of cocaine intake [12].

We know that morphine, cocaine, and methamphetamine have different pharmacological properties, and morphine analgesia and addiction are mainly related to opioid receptors. So, is HCN channel in VTA brain also involved in the regulation of morphine addiction behavior? This is an interesting issue in this study. Intra‐VTA infusion of ZD7288, a selective inhibitor of HCN channel, into rats with chronic morphine administration showed that the white box residence time of CPP experiment was significantly reduced compared with that before infusion of ZD7288. These results indicate that HCN2 channel plays an important role in morphine CPP. Combined with the recent studies on the involvement of HCN channels in cocaine and methamphetamine addiction, it is suggested that HCN channels in VTA contributed to the regulation and occurrence of addiction. As our results also showed that chronic morphine administration induced the increase of HCN2 expression in dopaminergic neurons of VTA in rats, these studies strongly suggest that dopamine reward pathway and HCN channel are closely related to addiction.

Our study still has some limitations as it cannot fully clarify the function and role of HCN2 in the occurrence of morphine addiction. This requires a systematic analysis of HCN subtype transgenic animals, video behavioral coding of addiction, and developmental biology. However, our research is still very valuable. It not only proves that chronic morphine administration can induce the upregulation of HCN2 subtype expression of dopaminergic neurons in VTA, but also suggests that HCN2 is involved in morphine addiction, which provides a new target for drugs to intervene morphine addiction and provides clues and basis for solving the problem of morphine addiction.

## Conflict of interest

The authors declare no conflict of interest.

### Peer review

The peer review history for this article is available at https://www.webofscience.com/api/gateway/wos/peer‐review/10.1002/2211‐5463.13888.

## Author contributions

JY performed multiple experiments, data acquisition and wrote the first draft of the manuscript, confirmed the authenticity of all raw data, and agreed to be accountable for all aspects of the study. YL and DL contributed to the design of the study, participated in revising the manuscript. CC participated in immunofluorescence experiments. XW contributed to the design of the study, participated in revising the manuscript. All authors have read and approved the final manuscript.

## Data Availability

The data that support the findings of this study are contained within the article.
